# Adaptation to visual or auditory time intervals modulates the perception of visual apparent motion

**DOI:** 10.3389/fnint.2012.00100

**Published:** 2012-11-05

**Authors:** Huihui Zhang, Lihan Chen, Xiaolin Zhou

**Affiliations:** ^1^Department of Psychology, Center for Brain and Cognitive Sciences, Peking UniversityBeijing, China; ^2^Key Laboratory of Machine Perception (Ministry of Education), Peking UniversityBeijing, China

**Keywords:** interval timing, adaptation, visual apparent motion, cross-modal interaction, Ternus display

## Abstract

It is debated whether sub-second timing is subserved by a centralized mechanism or by the intrinsic properties of task-related neural activity in specific modalities (Ivry and Schlerf, 2008). By using a temporal adaptation task, we investigated whether adapting to different time intervals conveyed through stimuli in different modalities (i.e., frames of a visual Ternus display, visual blinking discs, or auditory beeps) would affect the subsequent implicit perception of visual timing, i.e., inter-stimulus interval (ISI) between two frames in a Ternus display. The Ternus display can induce two percepts of apparent motion (AM), depending on the ISI between the two frames: “element motion” for short ISIs, in which the endmost disc is seen as moving back and forth while the middle disc at the overlapping or central position remains stationary; “group motion” for longer ISIs, in which both discs appear to move in a manner of lateral displacement as a whole. In Experiment 1, participants adapted to either the typical “element motion” (ISI = 50 ms) or the typical “group motion” (ISI = 200 ms). In Experiments 2 and 3, participants adapted to a time interval of 50 or 200 ms through observing a series of two paired blinking discs at the center of the screen (Experiment 2) or hearing a sequence of two paired beeps (with pitch 1000 Hz). In Experiment 4, participants adapted to sequences of paired beeps with either low pitches (500 Hz) or high pitches (5000 Hz). After adaptation in each trial, participants were presented with a Ternus probe in which the ISI between the two frames was equal to the transitional threshold of the two types of motions, as determined by a pretest. Results showed that adapting to the short time interval in all the situations led to more reports of “group motion” in the subsequent Ternus probes; adapting to the long time interval, however, caused no aftereffect for visual adaptation but significantly more reports of group motion for auditory adaptation. These findings, suggesting amodal representation for sub-second timing across modalities, are interpreted in the framework of temporal pacemaker model.

## Introduction

Timing is fundamental for the brain to process dynamically changing stimuli and interact with the environment. The neural system processes temporal information across a wide range of scales, from microseconds to circadian rhythms, with each scale corresponding to a specific underlying processing mechanism (Fraisse, 1963; Pöppel, 1988; Czeisler et al., 1999; Grothe, 2003). It has been revealed that sub-second timing is closely related to perceptual processing (Rammsayer, 1999; Wearden et al., 2007) and free of cognitive processing, although there is evidence showing that emotional arousal states, triggered by emotional stimuli such as emotional pictures, affect sub-second time perception in another modality (Shi et al., 2012). However, temporal processing above one second may involve more sophisticated cognitive processes (Rammsayer, 1999; Lewis and Miall, 2003; Mauk and Buonomano, 2004; Buhusi and Meck, 2005). The question remains as to whether sub-second interval timing in different modalities is subserved by a centralized mechanism (“central timer” or “central clock”; Grondin and Rousseau, 1991; Penton-Voak et al., 1996) or by the intrinsic properties of task-related neural activity in a particular modality (Ivry and Schlerf, 2008).

The traditional view toward sub-second temporal processing assumes that it is achieved by a centralized mechanism, independent of the specific sensory modality that conveys the temporal information. An implement of this idea is the “temporal pacemaker” model (Treisman, 1963; Treisman et al., 1990, 1994; Ivry et al., 2002), which consists of two major components. The first is a temporal oscillator that emits regular pulses at some fundamental frequency. These pulses are gated to a second component, a calibration or “gain control” or switch unit that can increase or decrease the frequency. The modulated pulses are counted and stored in working memory. In addition, temporal frequency of the repetitive, rhythmic stimuli could modulate the speed of pacemaker. Repetitive stimuli (clicks or flashes) of high temporal frequency may increase the speed of pacemaker, such that more pulses are accumulated in a given time; repetitive stimuli of low temporal frequency may decrease the speed of pacemaker, with less accumulated pulses for a given time (Ono and Kitazawa, 2011).

This model is supported by an increasingly large body of evidence. Firstly, psychophysics studies on visual and auditory sub-second time perception all showed that the ability to discriminate two time intervals is determined by the ratio of just-discriminable time difference to the base interval, suggesting that there might be a common temporal mechanism to compute the time information (i.e., the number of pulses; Creelman, 1962; Allan and Kristofferson, 1974; Divenyi and Danner, 1977; Killeen and Weiss, 1987; Keele and Ivry, 1991; Ivry, 1993). Secondly, tasks differed in sensorimotor processing (time perception vs. time reproduction) and in modality of stimuli used to define the intervals (visual vs. auditory) all showed a linear increase in performance variability as a function of the interval duration, and individuals' performances in tasks related to perception and reproduction of time intervals were highly correlated. These findings can be adduced to support the existence of a centralized internal clock which functions in all the tasks (Keele et al., 1985; Ivry and Hazeltine, 1995; Merchant et al., 2008). Thirdly, cross-modal adaptation experiments showed that adaptation to intervals defined by audiovisual events affect the perceived direction of visual apparent motion (AM) (Freeman and Driver, 2008); learning studies also demonstrated that training in a timing context can be generalized to other timing behaviors. For instance, learning to discriminate time intervals in the tactile domain can affect the performance in a similar task in the auditory domain (Nagarajan et al., 1998) and vice versa (Meegan et al., 2000). Such crossmodal transfer in timing suggests that there might be amodal representation and centralized time mechanism across different sensory modalities.

However, recent studies challenged this view. Using a direct visual temporal interval discrimination task, Lapid and Ulrich (2009) found no transfer of the learned time interval from the auditory to the visual domain. Using an adaptation paradigm, Becker and Rasmussen (2007) found a robust auditory temporal rhythm aftereffect, but only when the adaptation and test stimuli came from the same modality. In this study, an auditory test rhythm (400 ms interval) was preceded by an either faster or slower auditory rhythm and participants were asked to replicate the rhythm by pressing a button. They found a significant negative aftereffect, i.e., after adaptation to a faster rhythm, the reproduced rhythm was slower than the test rhythm; after adaptation to a slower rhythm, the reproduced rhythm was faster than the test rhythm. This aftereffect vanished when the test rhythm was presented in the visual modality. The authors suggested distinct mechanisms for sub-second temporal processing in different modalities.

A problem with Becker and Rasmussen (2007) is that reproduction of auditory rhythms is generally more accurate than that of visual rhythms (Welch and Warren, 1980; Glenberg et al., 1989; Glenberg and Jona, 1991; Recanzone, 2003). It is possible that the null crossmodal adaptation aftereffect with the visual stimuli in Becker and Rasmussen (2007) was due to the inaccuracy in perceiving time intervals conveyed by visual flashes. Alternatively, reproduction of visual rhythm is less reliable due to this motor activity being tightly coupled with inaccurate visual temporal processing (Repp, 2003; Patel et al., 2005). Thus, it might be the unreliable perception of visual rhythm and/or inaccurate motor reproduction of the visual rhythm, rather than the lack of cross-modal adaptation, that caused the null effect in the reproduction task.

**Figure 1:**
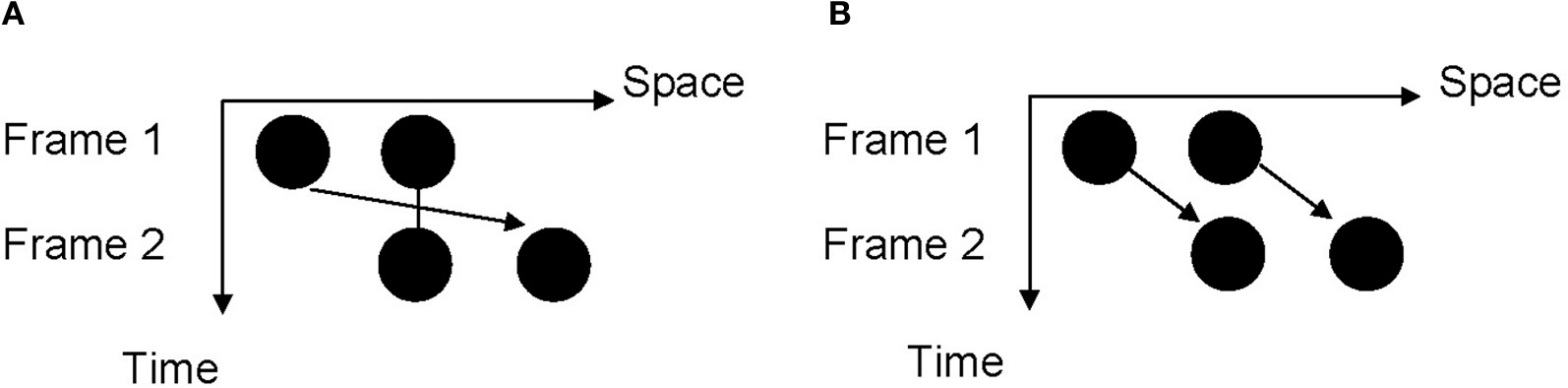
**Illustration of the Ternus display. (A)** “Element motion” percept: the disc which occupies the same position in two frames is perceived to remain static or to blink at the same location while the “outer” discs are perceived to move from one location to the other. **(B)** “Group motion” percept: the two discs are perceived to move together in the manner of a coherent lateral displacement.

To avoid the potential pitfalls associated with the reproduction task which *explicitly* measures the time interval processing, here we used the visual Ternus display to *implicitly* measure the processing of time intervals in the sub-second range (Figure 1). A typical Ternus display is composed of two frames with a variable inter-stimulus interval (ISI) (Ternus, 1926; Petersik and Rice, 2006; Shi et al., 2010). The first frame of the display contains two discs, and the second frame contains the same two discs with the second disc of the first frame and the first disc of the second frame sharing the same location. Depending on the locations of the first and the second frames, the AM could be either rightward or leftward. The Ternus display is an ambiguous display of which two different kinds of AM can be perceived depending on the ISI. At a short ISI, observers see the “overlapping” disc of two frames remaining stationary (or just blink) and the outer disc moving back and forth; this is called “element motion.” At a long ISI, observers see the discs of one frame moving as a whole; this is called “group motion.” The classification of two percepts of Ternus AM is a function of the ISI between the two frames, and we can use the report of element vs. group motion to measure the change of the implicitly perceived time interval triggered by temporal adaptation.

Specifically, we carried out three experiments (plus a control experiment) in which the ISI of the probe Ternus display was set at a time interval (about 125 ms) in which the report of element vs. group motion was equally probable (i.e., a bistable situation). The empirical question was whether (and how) this balance between the two types of percepts would be altered by the preceding adaptation procedures and whether different schemes of adaptation would differentially affect the perception of motion in the Ternus display. The adaptation scheme in Experiment 1 used visual Ternus displays in which the ISI between the two frames was set at either 50 ms (for element motion) or 200 ms (for group motion). If adaptation to the two time intervals is equally effective in affecting temporal processing, we would expect to observe more reports of group motion for the probe displays after adapting to the short ISI and more reports of element motion after adapting to the long ISI. Experiments 2 and 3 used, respectively, paired blinking discs and paired auditory beeps to demarcate the time intervals that the participants were supposed to adapt to. There were also two types of intervals for adaptation, 50 or 200 ms. Although the adaptation schemes in Experiments 1 and 2 were both presented in the visual modality, they differed in the extent to which the adaptation scheme was similar to the probe in perception and task structures. The adaptation schemes in Experiments 2 and 3 were similar in task structure, but differed in the presentation modality. If interval timing in the sub-second range relies on an amodal neurocognitive mechanism, we should observe an adaptation aftereffect not only for adaptation schemes sharing the modality with the probe (i.e., Experiments 1 and 2), but also for cross-modal adaptation schemes (i.e., Experiment 3); moreover, the pattern of the aftereffect should be similar across experiments, although the task structure may to a certain degree modulate this pattern. If, however, time intervals are encoded as intrinsic properties of stimulus processing, the earlier temporal processing at the adaptation stage should have different impacts upon (implicit) temporal processing at the probe stage, depending on the task structure and/or modality of adaptation schemes.

## Methods

### Participants

Seventeen students (mean age 21.9 years old, 8 females), nineteen (mean age 20.7 years old, 12 females), and twenty-five (mean age 20.7 years old, 14 females) from Peking University participated in Experiments 1, 2, and 3, respectively. Twenty seven students (mean age 20.6 years old, 13 females) participated a control experiment (Experiment 4). They all had normal or corrected-to-normal vision and normal hearing and were naïve to the purpose of the research. We used different pools of participants for the four experiments because we were concerned with the possible contaminations across different tasks: for example, participants could adopt response strategies if each participant takes part in all the experiments (as we observed in pilot studies but not reported here). Informed consent was obtained from each participant as required by the Ethics Committee, Department of Psychology at Peking University.

### Stimuli and apparatus

Each probe Ternus display was composed of two frames, with each frame of two black discs (12.71 cd/ m^2^ in luminance) presented horizontally on a gray background (16.11 cd/ m^2^ in luminance). The two frames shared one disc location at the center of the screen and contained the other two discs on the horizontally opposite side of the center (Figure 1). The diameter of each black disc was 1.6° in visual angle, and the distance between the centers of the two adjacent discs was 3.1°. The duration of each frame was 30 ms. The ISI that yielded equally probable reports of element motion and group motion was determined individually for each participant in a pretest (see “Procedure”).

For the three adaptation schemes, the Ternus display in Experiment 1 was structured in the same way as the probe display, except that the ISI between the two frames was set at either 50 or 200 ms. The time interval between the two paired blinking discs in Experiment 2 was also set at either 50 or 200 ms. The time interval between pairs of discs were 400 ms. Each disc had the same physical properties as the disc in the Ternus display. All the discs were presented consecutively at the center of the screen. The auditory beeps in Experiment 3 were presented binaurally, with the duration of each beep (65dB, 1000 Hz, sampled at 44.1 kHz for Experiment 3; 65 dB, 500 or 5000 Hz for control experiment) lasting 30 ms. Again, the time interval between the two paired beeps were either 50 or 200 ms and the interval between pairs of beeps was 400 ms.

The testing room was dimly lit with an average ambient luminance of 0.12 cd/m^2^. Visual stimuli were presented on a 22-inch CRT monitor (1,024 × 768 pixels; 100 Hz) positioned at eye level. Viewing distance was set to 57 cm, maintained by using a chin-rest. A headset (Philips, SHM 1900) was used to emit sound stimuli. Stimulus presentation and data collection were implemented by computer programs which was developed with Matlab 7.1 (MathWorks Inc., Natick, MA) and Psychophysics Toolbox (Brainard, 1997; Pelli, 1997).

### Procedure

Prior to the formal experiment, participants underwent practice to be familiar with a Ternus display of either typical “element motion” (ISI = 50 ms) or typical “group motion” (ISI = 200 ms) percept. They were asked to discriminate the above two percepts by pressing the left and right mouse button to indicate responses for “element motion” and “group motion,” respectively. The mapping between button and response type was counterbalanced across participants. When participants made an incorrect response, an immediate feedback appeared on the screen showing the percept (element motion or group motion) that should be reported. The practice session continued until the participant's accuracy of report was close to 100%. Almost all the participants could meet this standard within 120 trials. They then underwent the pretest which aimed to find out each participant's point of subjective equality (PSE), i.e., the time interval on which the probabilities of reporting “element motion” and “group motion” were equal (50% each).

#### Pretest

A typical visual Ternus display procedure was used. The ISI between the two visual frames of Ternus display was selected from one of the following six durations: 50, 80, 110, 140, 170, or 200 ms. Directions of AM (leftward or rightward) were balanced across trials. Each configuration (with ISI level and motion direction) was presented 40 times. All the 240 trials (6 levels × 40 trials) were randomized in presentation order. These trials were divided into 4 blocks. Participants could take a short break between blocks.

A trial started with a fixation cross presented on the center of the screen for 300 ms. Next, a blank display (with a gray background) was shown for a random duration of 300–500 ms to reduce time-based expectations toward the next stimulus. Then the Ternus display with a variable ISI (50, 80, 110, 140, 170, or 200 ms) was presented. After a blank of 300 ms, participants were presented with a question mark until they made a two-alternative forced choice response indicating whether they had perceived “element motion” or “group motion.” The inter-trial interval was 500 ms.

For each ISI condition, the percentage of “group motion” reports was collapsed over two motion directions. The six data points (one for each ISI) were fitted into the psychometric curve using a logistic function (Treutwein and Strasburger, 1999). The transitional ISI (PSE) at which the participant was equally likely to report the two percepts could be calculated by estimating the 50% of reporting “group motion” on the fitted curve. For each participant, we calculated his/her PSE immediately after the pretest session. The Ternus display with ISI equal to PSE would be used as a probe in the following adaptation session.

Comparisons were conducted for the PSEs derived for the three groups of participants. There were no significant differences between the “Visual-AM” (114.4 ± 13.8 ms), the “Visual-Blink” (117.1 ± 14.0 ms), and the “Beeps” (120.4 ± 13.0 ms) groups, *F*_(2, 58)_ = 1.00, *p* > 0.1. Comparisons were also made for the JNDs (just noticeable differences), which measured the task difficulty/participants' sensitivity of discriminating the two percepts in visual Ternus display. There were no differences between the three groups of participants (21.7 ± 5.2, 20.8 ± 6.0, and 22.2 ± 5.4 ms, respectively), *F*_(2, 58)_ = 0.33, *p* > 0.1. These results suggested that the three groups of randomly selected participants were well matched in their basic abilities in perceiving AM and in the implicit processing of time intervals between visual frames.

#### Adaptation

Each trial consisted of two phases: exposure and immediate probe test. In Experiment 1 (“Visual-AM”), the adaptation stimuli were Ternus displays of either typical “element motion” (short interval, ISI = 50 ms) or typical “group motion” (long interval, ISI = 200 ms). The probe test was a Ternus display with ISI equal to the PSE obtained in the pretest session, which rendered ambiguous percepts between “element motion” and “group motion.” The trials for two types of adapting stimuli (“element motion” and “group motion”) were arranged in blocks, the presentation order was pseudo-randomized. Each participant received 8 blocks (4 blocks for each adaptation type) with each block containing 20 target trials and 10 filler trials. We introduced filler trials with Ternus displays of typical “element motion” (ISI = 50 ms) or “group motion” (ISI = 200 ms) among probe trials to minimize potential response bias. The direction of Ternus AM (leftward or rightward) was same between exposure phase and probe test. For each trial, after a fixation of 300 ms, the exposure phase started. The exposure phase was composed of 7–9 repetitions of Ternus display. The time interval between consecutive presentations of the Ternus display was 400 ms, which was good enough to separate the adjacent adapting Ternus display clearly with a pilot test. After the presentation of adapting stimuli, followed by a 900 ms blank interval, the probe Ternus display was given. After a 1200 ms blank interval, a question mark appeared on the screen and remained until a two-alternative forced choice of either “element motion” or “group motion” was made. For each trial, participants were instructed to respond to the last presentation of Ternus display. The inter-trial interval was 500 ms. Participants could take a short break between blocks.

In Experiment 2 (“Visual-Blink”), the adapting time intervals (50 or 200 ms) were given by a sequence of two consecutively presented blink discs (the same central disc of Ternus display used in Experiment 1). Participants were asked to respond to the probe Ternus after viewing the blinking discs. The other arrangements of parameters and response method were the same as in Experiment 1.

In Experiment 3 (“Beeps”), the adapting time intervals (50 or 200 ms) were given by a sequence of paired beeps. During the exposure phase, participants were instructed to keep looking at the cross presented on the center of the screen while listening to the auditory beeps. This arrangement was used to make participants maintain their fixation on the location where the probe Ternus would be presented as in Experiment 1 and 2. Participants were asked to judge probe Ternus display after hearing the beeps. The other arrangements of temporal parameters and response method were the same as in Experiment 1.

In Experiment 4 (“Beeps”), the adapting time intervals (50 or 200 ms) were given by a sequence of paired beeps, different to Experiment 3 (auditory stimuli with fixed pitch: 1000 Hz), two pitches (one sequence of beeps were of the same pitches) were used: 500 or 5000 Hz. The procedures for adaptation and probe test were similar to those in Experiment 3, except that when the whole adaptation and the test probe trials were finished, participants took an additional subjective rating task, in which they were asked to rate on a 7-point Likert scale about the perceived degree of arousal for the following four types of auditory stimuli sequence: short interval-low pitch, short interval-high pitch, long interval-low pitch, long interval-high pitch. Each type was repeated three times and the presentation orders of the above types of sound sequences were randomized.

## Results

For filler trials in all the four experiments, the average proportion of reporting “group motion” was less than 10% of the filler trials with a ISI of 50 ms (“element motion” displays) and more than 90% of the filler trials with a ISI of 200 ms (“group motion” displays), indicating that participants generally had clear percepts of element motion and group motion. Each individual's performance accuracy on the filler trials were within the distribution of mean value for all the participants plus/minus three standard deviations.

**Figure 2:**
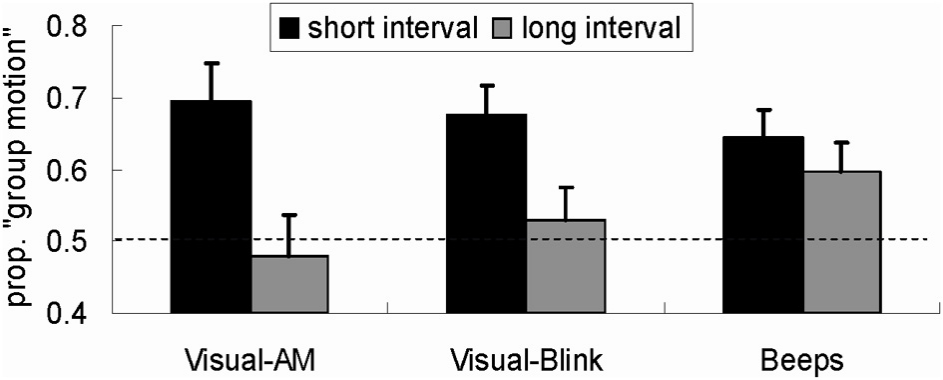
**Reports of group motion for the probe Ternus display (with ISI = the time corresponding to PSE) for the three experiments.** The values in Y-axis represent the proportion of “group motion.” The black bars represent “group motion” reports after short time intervals (50 ms) adaptation and the gray bars represent “group motion” reports after long time intervals (200 ms) adaptation. “Visual-AM,” “Visual-Blink,” and “Beeps” refer to, respectively, the three experiments in which the visual Ternus apparent motion, visual blinking discs and auditory beeps were used in different adaptation schemes. The error bar represents one standard error.

We made statistical comparisons between Experiments 1–3 (Figure 2). For the critical trials, analysis of variance (ANOVA) was conducted, using the difference between the proportion of “group motion” report and the proportion (0.50) corresponding to the PSE as the dependent measure and with experiment as a between-participant factor. The main effect of time interval was significant, *F*_(1, 58)_ = 29.99, *p* < 0.001, with more reports of group motion after the adaptation to the short time interval (17.1%) than after adaptation to the long time interval (3.5%). Further tests showed that while overall the adaptation effect was significant for the short interval, *F*_(1, 58)_ = 44.99, *p* < 0.001, it was not for the long time interval, *F*_(1, 58)_ = 1.63, *p* > 0.1.

Importantly, the interaction between adaptation scheme time and experiment was significant, *F*_(2, 58)_ = 4.074, *p* < 0.05, indicating that the adaptation schemes had different impacts upon the report of group motion in different experiments. Further analysis was conducted to examine the interaction in detail. We first tested the adaptation aftereffect for the short or the long time interval, respectively, treating experiment as a between-subjects factor. This test found no significant differences between experiments for either the short interval adaptation, *F*_(2, 58)_ = 0.35, *p* > 0.1, or the long interval adaptation, *F*_(2, 58)_ = 1.65, *p* > 0.1. However, separate comparisons with 0.5 showed that all short interval adaptations in different experiments led to more reports of group motion, *ps* < 0.01; for the long interval adaptation, the aftereffect was not observed in Experiments 1 and 2 with visual modality, *ps* > 0.1, but in Experiment 3 with auditory adaptation, *p* < 0.05.

On the other hand, comparison between the adaptation effects after the short and long time interval adaptation in each experiment revealed a significant difference in Experiment 1, *F*_(1, 58)_ = 21.62, *p* < 0.001, in Experiment 2, *F*_(1, 58)_ = 10.86, *p* < 0.01, but not in Experiment 3, *F*_(1, 58)_ = 1.44, *p* > 0.1.

In Experiment 4, the averaged appraisal scores are: high pitch-short interval (5.8), high pitch-long interval (4.9), low pitch-short interval (5.2), low pitch-long interval (4.0). The main effect of pitch was significant, *F*_(1, 26)_ = 12.676, *p* < 0.01; The main effect of interval was also significant, *F*_(1, 26)_ = 59.297, *p* < 0.001. The auditory signals with higher pitch triggered more arousal (5.4) than the low pitch did (4.4). The sequences with shorter inter-intervals induced more arousal (5.4) than those with longer inter-intervals (4.4). However, the interaction between pitch and interval was not significant, *F*_(1, 26)_ = 1.040, *p* > 0.1. For reports of the proportion of “group motion,” both the main effects of pitch [*F*_(1, 26)_ = 0.076, *p* > 0.1] and interval [*F*_(1, 26)_ = 1.649, *p* > 0.1] were not significant, and the interaction was also not significant, *F*_(1, 26)_ = 1.342, *p* > 0.1. *Post-hoc* T tests revealed that the percentages of “group motion” percentages, were significantly larger than 0.5 in all the four sub-conditions (short-high, *p* < 0.01; short-low, *p* < 0.01; long-high, *p* < 0.01; long-low, *p* < 0.05).

## Discussion

Using a temporal adaptation paradigm, we demonstrated that adaptation to the preceding short temporal interval (50 ms) induced significant negative aftereffects on perception of the subsequent visual Ternus AM, irrespective of whether the time interval was conveyed by events in the same modality (i.e., visual AM or blinking discs) or in a different modality (i.e., auditory beeps). This pattern of aftereffects suggests that there is a general “temporal pacemaker” mechanism (Treisman, 1963; Treisman et al., 1990, 1994) and amodal representation for sub-second interval time. Although adaptation to the preceding long temporal interval (200 ms) did not lead to unanimous significant aftereffects across the three tasks, the differences between experiments may reflect the differential impacts of temporal attending (see below) in the visual and auditory modalities, rather than distinct time interval representations in different modalities for the sub-second range.

The within-modality aftereffect for the short time interval adaptation replicated Becker and Rasmussen (2007); the significant between-modality adaptation aftereffect, however, contrasted sharply with the null effect in Becker and Rasmussen (2007), suggesting that the implicit task used here is more sensitive to the adaptation aftereffect than the explicit reproduction task. The existence of cross-modality adaptation effect is clearly inconsistent with the idea of distinct timers for different modalities (Keele et al., 1989; Ivry, 1996; Pashler, 2001), at least at the sub-second range. Instead, it suggests that there is amodal representation of internal clock and adapting to the repetitive stimuli in one modality can alter the speed of the internal clock, leading to a subjectively changed percept of the subsequent time interval in another modality. Specifically, according to the “temporal pacemaker” model, temporal frequency of preceding repetitive stimuli can influence the speed of internal clock and hence the perceived subsequent (target) time interval. A higher frequency can increase the speed of internal clock, rendering a given time interval being perceived longer; a lower frequency can decrease the speed of internal clock, making a given time interval being perceived shorter (Ono and Kitazawa, 2011).

On the other hand, the regular repetitive, rhythmic stimuli can trigger temporal attending—a shift of attentional focus to anticipate the onsets of subsequent events (Jones et al., 2002). In other words, the temporal attending mechanism, established after exposing to either visual or auditory sequences, guides the distribution of attentional resources around the time points the rhythmic stimuli are presented. The pattern of temporal distribution of attentional resources over different time points can be applied to subsequent within-modal or cross-modal events, affecting the temporal processing of these events (Jones, 1976; Large and Jones, 1999; Jones et al., 2002). This effect of temporal attending is dependent on the reliability of perceiving the temporal regularity. Given that perception and reproduction of auditory rhythmic sequences are generally better than perception and reproduction of visual rhythmic sequences (Welch et al., 1986; Glenberg et al., 1989; Glenberg and Jona, 1991; Recanzone, 2003, 2009; Repp, 2003; Patel et al., 2005), it is possible that the effect of temporal attending is more potent in the auditory domain than in the visual domain.

We suggest that the change of speed of the internal clock by the repetitive adaptation stimuli with short or long time intervals and the efficiency of temporal attending in different modalities co-determined the patterns of adaptation aftereffects. For the short interval (50 ms) adaptation, the internal clock speed was accelerated by both visual and auditory adaptation stimulus sequences, potentially leading to more reports of group motion in the subsequent Ternus displays. However, the temporal attending, established after exposing to either visual or auditory sequences, affected the distribution of attentional resources around the time points that the two visual frames of the Ternus display were presented (Aydin et al., 2011). Specifically, although the first frame could be aligned with the first time point of the temporal attending, the second frame, located after the second time point of the temporal attending, could be “pulled” in time closer to the second time point (see Aydin et al., 2011; Keetels et al., 2007; Chen et al., 2010; Shi et al., 2010 for the effect of temporal attention on perceptual segregation), potentially leading to more reports of element motion. Thus, adaptation to rhythmic sequence of visual or auditory events had two potentially conflicting consequences; with the short interval (50 ms), the increase of clock speed could play a dominant role, leading to more reports of group motion overall.

For the long interval (200 ms) adaptation, the slowed-down clock would make the interval between the frames of the visual display being perceived shorter, and this should potentially lead to more reports of element motion. However, the temporal attending mechanism would “pull” the second frame, located before the second time point of temporal attending, toward the second point, potentially leading to more reports of group motion. These two conflicting effects could cancel each other for adaptation within the visual modality. For cross-modality adaptation, however, given that adapting to rhythmic auditory events could activate a stronger temporal attending mechanism than the adapting to rhythmic visual events (Welch et al., 1986; Jones et al., 2002; Recanzone, 2003, 2009), the overall effect was the stronger segregation of the two Ternus frames and more reports of group motion.

**Figure 3:**
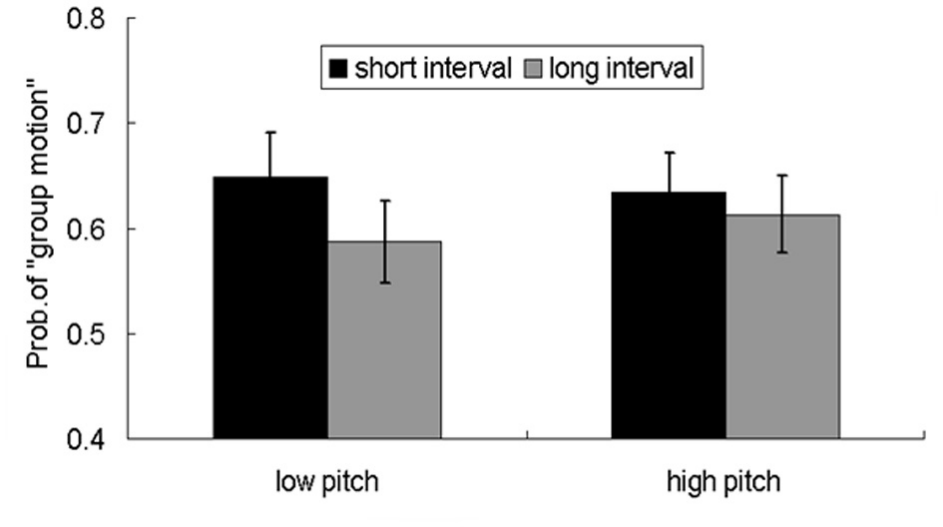
**Reports of group motion for the probe Ternus display after adapting to the four kinds of auditory sequences: low pitch-short interval, low pitch-long interval, high pitch-short interval, and high pitch-long interval.** The error bar represents standard error.

The finding of equivalent aftereffects for auditory beeps with short and long intervals in cross-modal adaptation is surprising. To replicate this effect and to rule out an alternative account which attributes the positive aftereffects to arousal evoked by auditory input, we conducted a further experiment similar to Experiment 3 except that the pitch of the auditory beeps was manipulated. Previous studies showed that the arousal state correlates with the fundamental frequency of sound and temporal rhythms (Banziger and Scherer, 2005; Bruck et al., 2008, 2009). We adopted a within-subject factorial design, with two levels of adaptation time intervals (50 or 200 ms) and two types of pitches (500 or 5000 Hz), plus an additional subjective rating task of perceived arousal (Edelman, 1970; Gudjonsson, 1981; Slomine et al., 1999; Cuthbert et al., 2000). The subjective ratings of the perceived arousal were differed among the four types of auditory sequences (short interval-low pitch, short interval-high pitch, long interval-low pitch, long interval-high pitch), both auditory sequences with higher pitch and short intervals were perceived as higher arousal. However, the perceived different arousal levels did not affect the pattern of adaptation aftereffects, both short interval and long interval adaptation lead to more reports of “group motion” and there was no statistical difference for the percentages of “group motion” in the two conditions (Figure 3). The less impact of arousal upon the temporal adaptation aftereffect might be due to three possible reasons: *First*, there is interplay between attention and arousal. The arousal effects are two sides of a coin. The (higher) arousal contributes to accumulate more pulses as implicated in the pacemaker model (Treisman, 1963), the perceived interval would be expanded and hence give rise to a dominant percept of “group motion”; on the other hand, the arousal auditory stimuli attract more attention, meanwhile less attention resources were allocated to time processing itself. The reduced attention on the pacemaker would lead to a loss of pulses and reduced interval, as indicated by a number of relevant studies (Fortin, 2003; Buhusi and Meck, 2006; Noulhiane et al., 2007; Buhusi and Meck, 2009). The reduced interval would give rise to dominant percept of “element motion”. The above opposite influences interact and cancel out, imposing non-observable impact on the probe visual motion. *Second*, adaptation to temporal intervals with short temporal length (about average 4.5 seconds for an adaptation trial in our experiments) might trigger an “immediate” arousal (Coull, 1998; Del-Fava and Ribeiro-do-Valle, 2004), and the effect of the arousal perhaps dissipates after several hundred milliseconds (with 900 ms delay between the offset of the adaptation sequence and the probe in current study) (Ulrich and Mattes, 1996; Fernandez-Duque and Posner, 1997; Coull et al., 2000). *Third*, even the arousal effect still remains after a temporal delay, the arousal effect has been revealed to be less important and somehow inhibited by the entrained attention issued by the auditory sequence (Jones et al., 2002; Del-Fava and Ribeiro-do-Valle, 2004). Previous study using visual discrimination tasks, where auditory stimuli as (preceding) accessory stimuli, would speed up the response to a subsequent visual stimulus, however, for the accessory auditory stimuli, the expectancy (of temporal attention) is revealed to be more important and could inhibit the “immediate arousal” effect (Del-Fava and Ribeiro-do-Valle, 2004). This analogous mechanism might operate in the current investigation using short temporal range for adaptation.

To conclude, using an adaptation paradigm with implicit test of timing, the present study found that adapting to a short time interval conveyed by either visual or auditory stimuli leads to more report of group motion in the subsequent visual Ternus probes; adapting to a longer time interval, however, caused no aftereffect for visual adaptation but significantly more reports of group motion for auditory adaptation. These results suggest that there exists amodal representation for sub-second timing, but adaptation to repetitive, rhythmic sequence of stimuli in different modalities may elicit temporal attending of different strengths, affecting the manifestation of adaptation after effects.

### Conflict of interest statement

The authors declare that the research was conducted in the absence of any commercial or financial relationships that could be construed as a potential conflict of interest.
